# Tumour‐derived exosomal miR‐3473b promotes lung tumour cell intrapulmonary colonization by activating the nuclear factor‐κB of local fibroblasts

**DOI:** 10.1111/jcmm.15411

**Published:** 2020-05-25

**Authors:** Cancan Du, Xixi Duan, Xiaohan Yao, Jiajia Wan, Yanru Cheng, Yuan Wang, Yan Yan, Lijing Zhang, Linyu Zhu, Chen Ni, Ming Wang, Zhihai Qin

**Affiliations:** ^1^ Medical Research Center The First Affiliated Hospital of Zhengzhou University Zhengzhou China; ^2^ School of Basic Medical Sciences The Academy of Medical Sciences of Zhengzhou University Zhengzhou China; ^3^ Key Laboratory of Protein and Peptide Pharmaceuticals Institute of Biophysics Chinese Academy of Sciences CAS‐University of Tokyo Joint Laboratory of Structural Virology and Immunology University of the Chinese Academy of Sciences Beijing China

**Keywords:** exosomes, fibroblasts, lung cancer, miR‐3473b, *Nfkbid*

## Abstract

Tumour‐derived exosomes have been shown to induce pre‐metastatic niche formation, favoring metastatic colonization of tumour cells, but the underlying molecular mechanism is still not fully understood. In this study, we showed that exosomes derived from the LLC cells could indeed significantly enhance their intrapulmonary colonization. Circulating LLC‐derived exosomes were mainly engulfed by lung fibroblasts and led to the NF‐κB signalling activation. Further studies indicated that the exosomal miR‐3473b was responsible for that by hindering the NFKB inhibitor delta's (NFKBID) function. Blocking miR‐3473b could reverse the exosome‐mediated NF‐κB activation of fibroblasts and decrease intrapulmonary colonization of lung tumour cells. Together, this study demonstrated that the miR‐3473b in exosomes could mediate the interaction of lung tumour cells and local fibroblasts in metastatic sites and, therefore, enhance the metastasis of lung tumour cells.

## 1 INTRODUCTION

Lung cancer is one of the most common malignant tumours with the highest morbidity and mortality rate.^1^ More than 70% of lung cancer patients' death is caused by metastasis. And the common metastatic sites of lung cancer are the lung, brain, liver, bone and adrenal gland.^2^ Although there is the possibility of multiple primary lung cancer (MPLC), patients with more than one tumour lesions are generally considered as metastasis.^3^ Early treatment is important to reduce lung cancer‐associated metastasis and death. However, in several cases, the primary lung cancer has already metastasized prior to initial diagnosis. Recent evidence suggests that prior to metastasis, tumour cells can induce pre‐metastatic niche (PMN) formation by altering the environment of distant tissues, which provides a suitable condition for tumour cells colonization, a key step of tumour metastasis.^4^, ^5^ Tumour‐derived exosomes, which potentially travel from primary tumour site to a new metastatic site, act as a potential mediator for establishing PMN,^4^, ^6^ but the underlying mechanisms are not fully understood. Therefore, exploring the molecular mechanisms of lung tumour‐derived exosomes mediate the PMN formation is crucial for developing targeted treatment of lung cancer metastasis in the future.

Exosomes are lipid bilayer‐encapsulated microparticles (30‐100 nm) released by various types of cells, which contain various proteins, lipids, DNA and miRNAs.^7^, ^8^, ^9^, ^10^ It has been illustrated that exosomes are critical factors in mediating the communication between tumour cells and stromal cells, and exosomes play an important role in promoting tumour growth, angiogenesis, immune escape and metastasis.^11^, ^12^, ^13^, ^14^, ^15^ For example, pancreatic cancer‐derived exosomes contain high amount of macrophage migration inhibitory factor (MIF), which recruits macrophages to induce the formation of PMN in the liver and promote liver metastasis.^6^ Except proteins, a large amount of exosomal miRNAs also play key roles in regulating tumour progression.^16^, ^17^ The primary cell‐derived exosomes were absorbed by receipt cells, and the released miRNAs regulate the expression of target genes at the post‐transcriptional level by binding to the 3′UTR sites of target mRNAs.^18^ For instance, miR‐3473b blocked the suppressor of cytokine signalling 3 (SOCS3) gene expression in BV2 microglial cells, leading to the increased pro‐inflammatory cytokines secretion in middle cerebral artery occlusion (MCAO) mice model.^19^ However, how lung tumour‐derived exosomal miRNAs mediate the PMN and metastatic colonization formation is still not fully understood.

Tumour‐associated fibroblasts, the main stromal cell components in various solid tumours, are believed to be involved in all stages of tumour progression.^20^, ^21^, ^22^ For example, exosomal miR‐155‐5p mediates the polarization of cancer‐associated fibroblasts (CAFs), which results in melanoma angiogenesis.^23^ Hepatocellular carcinoma (HCC)‐derived exosomal miR‐1247‐3p could accelerate HCC lung metastasis by leading to the β1 integrin‐mediated nuclear factor‐κB (NF‐κB) activation and pro‐inflammatory cytokines production in fibroblasts.^24^ However, the relevance between lung tumour‐derived exosomal miRNAs and fibroblast‐mediated lung tumour metastasis has not been investigated.

In this study, we explored how lung tumour‐derived exosomes promote LLC intrapulmonary colonization by communicating with local fibroblasts. Our data show that miR‐3473b contained in LLC‐derived exosomes plays a vital role in NF‐kB activation and inflammatory cytokines production in fibroblasts. The interaction between lung tumour‐derived exosomes and fibroblasts elucidates a new mechanism of intrapulmonary colonization, providing potential therapeutic targets for lung cancer.

## 2 MATERIALS AND METHODS

### 2.1 Cell lines and antibodies

Lewis lung carcinoma (LLC) cell line was provided by Professor Li Yan from Academy of Military Medical Sciences. Cells were cultured in Dulbecco's modified Eagle's medium (DMEM; HyClone), supplemented with 10% foetal bovine serum (FBS), and 100 IU/mL penicillin/streptomycin (P/S; Gibco). All cells were maintained at 37°C in a humidified atmosphere of 5% CO_2_. Details of all antibodies were listed in Table S1.

### 2.2 Isolation and identification of exosomes

The exosomes isolation was performed following standard protocol by ultracentrifugation.^25^ Namely, LLC cells were cultured in 10 cm dishes, which were grown to 80% confluence, washed thrice with phosphate buffered saline (PBS), replaced with 10% exosome‐free serum and cultured for 24 hours. The supernatant was centrifuged at 200 *g* for 10 minutes to discard the pellet and then at 20 000 *g* for 20 minutes to discard the pellet. The obtained supernatant was centrifuged at 100 000 *g* for 70 minutes, and the obtained exosomes were further purified to remove the protein content in PBS implementing the same ultracentrifugation conditions. Ultracentrifugation was done using an ultracentrifuge (HITACHI; CS150FNX). Exosomes were observed using the transmission electron microscope HT7700 (HITACHI).

### 2.3 Western blot

Cell and exosome lysates were prepared in RIPA lysate (Solarbio, R0020). Protein lysates were quantified using the Protein BCA kit (Thermo Fisher, #23228). Then, 30 µg protein was added to SDS‐PAGE gel, and transferred to a PVDF membrane. Primary antibody was incubated overnight at 4°C. Horseradish peroxidase‐conjugated secondary antibody was incubated at room temperature for 1 hour. Further, the membrane was tested with the ChemiDoc MP imaging system (Bio‐Rad). Western blot antibodies were listed in Table S1. Each test was carried out in triplicate.

### 2.4 Isolation of primary lung fibroblasts

For primary lung fibroblasts isolation, 6 weeks C57BL/6 female mice were killed. The harvested lungs were minced and incubated with 0.1 mg/mL collagenase IV (Sigma, C5138) for 30 minutes at 37°C. The cell suspension was filtered through a 70 μm filter, and the suspended cells were cultured in DMEM. The medium was changed the next day leaving behind adhered fibroblasts.

### 2.5 Exosomes tracing experiment

For exosomes labelling, exosomes were added to Cy5.5 (Fanbo Biochemicals, #1056), stained for 30 minutes at 37°C, following washed in PBS and centrifuged at 100 000 *g* for 70 minutes. Labelled exosomes were added to primary fibroblasts, in vitro, for 3 hours; then, nuclei were stained with DAPI. Confocal microscopy (PerkinElmer, Ultra VIEW VOX) was used to observe the whereabouts of exosomes. For in vivo experiments, labelled exosomes were intravenously injected into C57BL/6 female mice for 12 hours; then, lungs were harvested and single cells were isolated and stained with CD45 and fibroblast‐specific protein (FSP). For Immunofluorescence, frozen sections were prepared and stained with α‐smooth muscle actin (α‐SMA). The staining was observed by a Perkin Elmer, Vectra machine. Each test had three repeats.

### 2.6 Animal experiment

To investigate the role of exosomes in lung metastasis, 0.5 mg/kg LLC‐derived exosomes or PBS was injected into C57BL/6 female mice every 3 days via the tail vein. After three injections of exosomes, 5 × 10^5^ LLC cells were intravenously injected into mice at day 0; Additional 3 more times, exosomes were injected at day 0, 3, and 6, respectively. Mice were sacrificed, and lung metastasis was observed by HE staining at day 22. The percentage of immune cells was analysed by flow cytometry. One of the three independent experiments is represented, n = 5. To detect the effect of miR‐3437b on lung metastasis, exosomes or 0.5 mg/kg of formulated miR‐3473b mimic or inhibitor, or liposome alone was injected to mice every 3 days through tail vein. Similarly, three more times exosomes were injected after LLC transplanted. At day 22, mice were sacrificed and lung metastasis was observed by HE staining. The percentage of B cells was analysed by flow cytometry. One of the three independent experiments is represented, n = 4. The details of injection are shown in figures. All animal experiments were approved by the University Committee on Use and Care of Animals of Zhengzhou University.

### 2.7 Flow cytometry analysis

Mice were anesthetized with 5% chloral hydrate. The lungs were minced and incubated with 0.1 mg/mL collagenase IV (Sigma, C5138) for 30 minutes at 37°C. The suspension was filtered through a 70 μm filter and stained with the following antibodies: CD45‐Alexa Fluor700, B220‐APC, CD8‐PerCP, CD4‐APC/Cy7, Ly6G‐APC and CD11b‐FITC. Analysis was performed using flow cytometer (BD Canto), and data were analysed with FlowJo X (Tree Star). Each test was repeated three times.

### 2.8 Immunofluorescence

For immune cells staining, lung paraffin sections were stained with rabbit anti‐CD4, CD8 and CD19 antibody respectively. Peroxidase‐conjugated secondary antibody (CWBIO, #CW2035S) was used. Sections were visualized with DAB and counterstained using haematoxylin. For exosomes tracking and other staining, the harvested lung tissues were embedded in OCT. The frozen sections were fixed by cold methanol and incubated with primary antibodies, namely p‐p65 and α‐SMA. Alexa Fluor 488 donkey anti‐rabbit or goat IgG was used as the secondary antibodies. Images were captured with a Perkin Elmer, Vectra machine. Each test had three repeats.

### 2.9 Agarose nucleic acid electrophoresis

To verify the presence of miRNA in exosomes, 1.5% agarose gel was prepared. LLC‐secreted exosomal RNA was added into the gel, and LLC total RNA was used as the positive control. The electrophoresis band and its position were observed using the ChemiDoc MP imaging system (Bio‐Rad), and the size of the amplified product was compared with a nucleic acid molecular weight standard marker. Each test was carried out in triplicate.

### 2.10 Microarray analysis

Exosomal miRNAs microarray analysis was performed at Oebiotech Technology Co, Ltd., using the Agilent mouse miRNA microarray (Release 21.0, 8 × 60 K, Design ID: 070155). The sample labelling, microarray hybridization and array washes process were executed according to manufacturer's protocols (Agilent Technologies Inc.). Data analyses were performed using Feature extraction software (version 10.7.1.1, Agilent Technologies).

### 2.11 RNA sequencing

The gene expression of fibroblasts treated with LLC‐derived exosomes was determined using RNA sequencing. Briefly, two independently treated fibroblasts were collected and RNA samples were prepared for RNA sequencing. Illumina RNA sequencing was performed by OE biotech Co., Ltd. (Shanghai, China) using the Illumina HiseqTM 2500 (Illumina, Inc.). EdgeR software was used to analyse the data, and statistical significance was defined as *P* values lower than .05.

### 2.12 RNA extraction and real‐time PCR

Trizol reagent (Life Technologies) was used to extract Total RNA from cells. Reverse transcription was performed with PrimeScript™ RT Master Mix (TaKaRa). Real‐time PCR was performed using SYBR Green PCR Master Mix (Applied TaKaRa, Otsu) on an ABI PRISM 7300HT Sequence Detection System (Applied Biosystems). miRNA was extracted from exosomes using the MiRNeasy Mini Kit (Qiagen). Reverse transcription and qRT‐PCR of exosomal miRNAs were performed using miRNA first‐strand cDNA synthesis (stem loop method) and the miRNA fluorescent quantitative PCR kit (dye method), respectively. All RNA isolation and PCR experiments are according to the manufacturer's instructions. The relative expression levels of mRNA were calculated using the 2^−ΔΔ^ *^C^* ^t^ method. The sequences of all indicated primers were listed in Table S2. Each test had three repeats.

### 2.13 RNA interference

FITC‐labelled mimics and inhibitors of miR‐3473b, miR‐5119 and miR‐7005‐5P and their negative controls were purchased from Shanghai Biotechnology Corporation. The sequences of miRNA inhibitor and mimics were listed in Table S3 and Table S4. Transfection of siRNA and miRNA were completed using the LipoHigh liposome efficient transfection reagent (Shanghai Biotechnology Corporation), according to the manufacturer's instructions.

### 2.14 Statistical analysis

Statistical analysis was performed using GraphPad Prism software. All experiments were performed three times, and data were expressed as the mean ± SD. Student's *t* test was used to assess statistical significance. Statistically significant differences are indicated as follows: *P < .05; **P < .01.

## 3 RESULTS

### 3.1 Fibroblasts engulf LLC‐derived exosomes in vivo and in vitro

**Figure 1:**
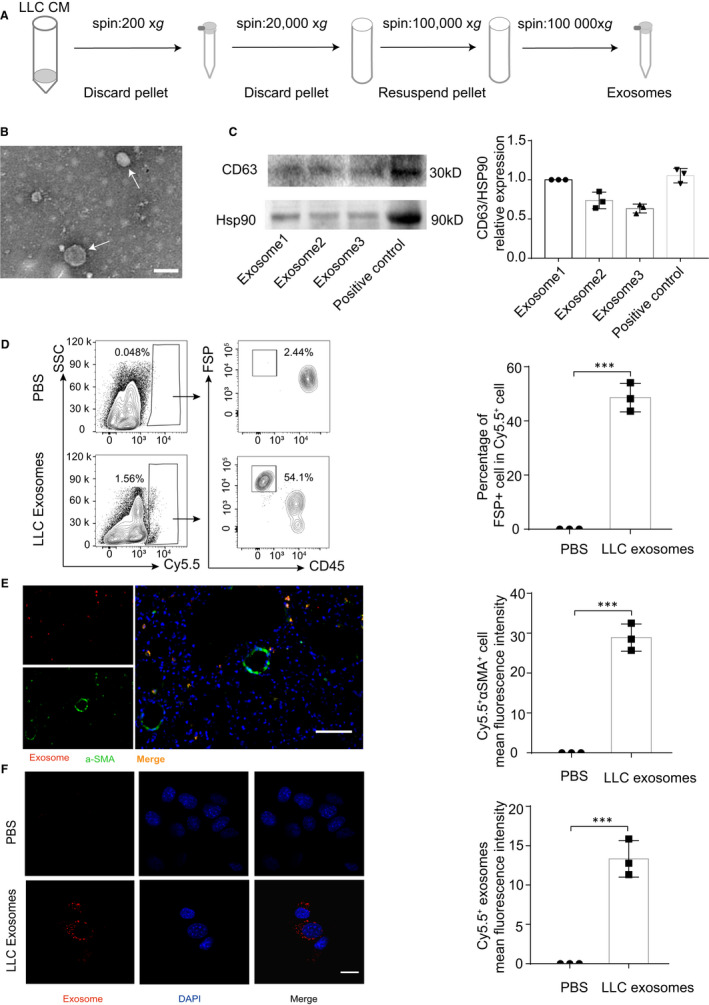
Fibroblasts engulf LLC‐derived exosomes in vivo and in vitro. A, Exosomes were separated from the LLC culture medium by ultracentrifugation, and the details were shown. B, Exosomes isolated from LLC were detected by electron microscopy. White arrows represented particles in typical exosome size. Scale bar, 100 nm. C, The expressions of CD63 and HSP90 in LLC exosomes were evaluated by Western blot and densitometry analysis, and foetal bovine serum protein was used as a positive control. D, Flow cytometry was used to detect the incorporation of Cy5.5‐labelled exosomes in FSP^+^ cells in lungs, and the percentage of Cy5.5^+^FSP^+^ cells were assessed. E, Immunofluorescence staining was used to examine the location of Cy5.5‐labelled exosomes and αSMA^+^ fibroblasts. Cy5.5^+^αSMA^+^ cells were quantitatively analysed, and representative images were shown. Scale bar, 50 µm. F, Cy5.5‐labelled exosomes were co‐cultured with primary lung fibroblasts for 3 h, and confocal microscopy was used to examine the uptake of exosomes. Cy5.5^+^ LLC exosomes in fibroblasts were quantitatively analysed, and representative images were shown. Scale bar, 10 µm. Exposure times for all slides were optimized using the unfixed slide. Each experiment was performed in triplicate, and data are presented as mean ± SD. Student's *t* test was used to analyse the data (***P < .001)

To detect the secretion of exosomes in lung cancer cells, we isolated exosomes from the LLC culture medium by ultracentrifugation (Figure 1A). The cup‐shaped structure and size of the isolated exosomes were identified using electron microscopy (Figure 1B). In addition, the expression of exosome markers Hsp90 and CD63 was verified (Figure 1C). To investigate the possibility of exogenous exosomes could be enriched and absorbed in the lung, we injected cy5.5‐labelled LLC‐derived exosomes into the tail vein of mice. Flow cytometry results showed that 54.1% of Cy5.5‐positive cell were CD45^‐^FSP^+^ Cell. Our previous study found that FSP is not only expressed in fibroblasts, but also by some macrophages.^26^ Nevertheless, CD45 staining negative excluded the possibility of macrophages. (Figure 1D). Immunofluorescence results further confirmed that LLC‐derived exosomes could be phagocytized by α‐SMA^+^ fibroblasts (Figure 1E). In addition, Cy5.5‐labelled LLC‐derived exosomes could be taken up by primary isolated lung fibroblasts (Figure 1F). Moreover, LLC‐derived exosomes slightly enhanced the proliferation capability of fibroblasts (Figure S1A,B).

### 3.2 LLC‐derived exosomes promote tumour cells colonization

**Figure 2:**
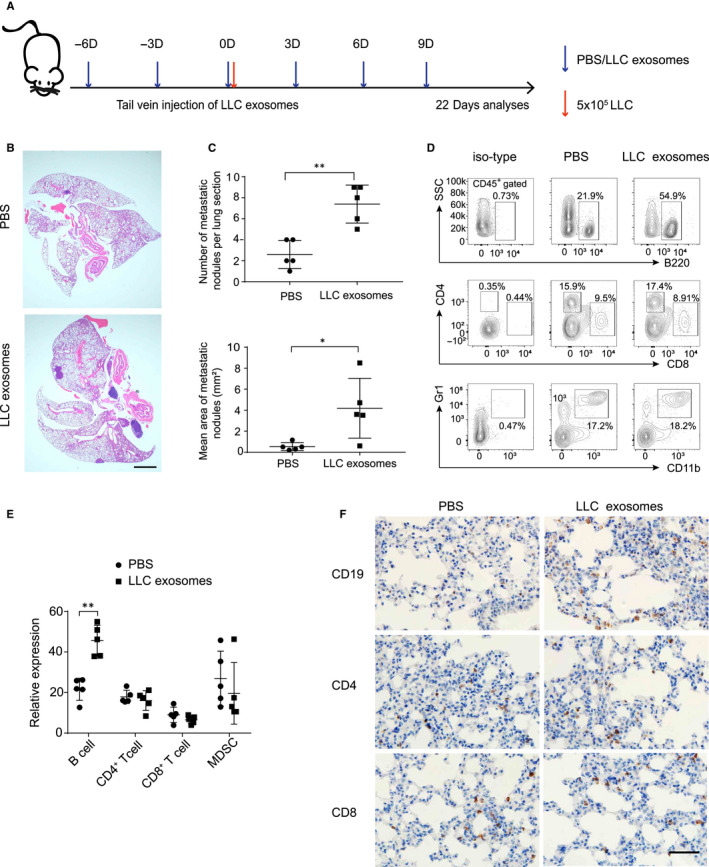
LLC‐derived exosomes could promote tumour cells colonization. A, Schematic diagram of exosomes treated animal experiment (n = 5). B, Representative H&E images of experimental lung sections in indicated groups. Scale bar, 1000 µm. C, Summarized the numbers and areas of lung metastasis in indicated groups. D and E, The proportion of immune cells in lungs were detected by flow cytometry. F, Representative images of mouse lung tissues stained with CD19, CD4 and CD8. Scale bar, 50 µm. Each experiment was performed in triplicate, and data are presented as mean ± SD Student's *t* test was used to analyse the data (*P < .05, **P < .01)

To explore the role of LLC‐derived exosomes in lung tumour seeding and colonization, we intravenously injected exosomes into mice through the tail vein before and after LLC cells transplantation (Figure 2A). HE staining showed that the numbers and areas of lung metastasis in the LLC‐derived exosomes group were increased (Figure 2B,C). This indicated that LLC‐derived exosomes could promote the colonization of LLC cells in lung. To further understand the metastatic microenvironment of lung, we analysed the population of immune cells in the lung. We found that the percentage of B220^+^ B cell was significantly increased in LLC‐derived exosomes group, whereas the numbers of CD4^+^, CD8^+^ T cells and CD11b, Gr1 marked MDSC showed no difference (Figure 2D,E). The result of immunohistochemistry further confirmed the increase of B cells in LLC‐derived exosomes group (Figure 2F). Taken together, these results indicate that LLC‐derived exosomes are able to induce the intrapulmonary colonization of lung tumour cells.

### 3.3 LLC‐derived exosomes activate the NF‐κB signalling pathway in fibroblasts by down‐regulating *Nfkbid*

**Figure 3:**
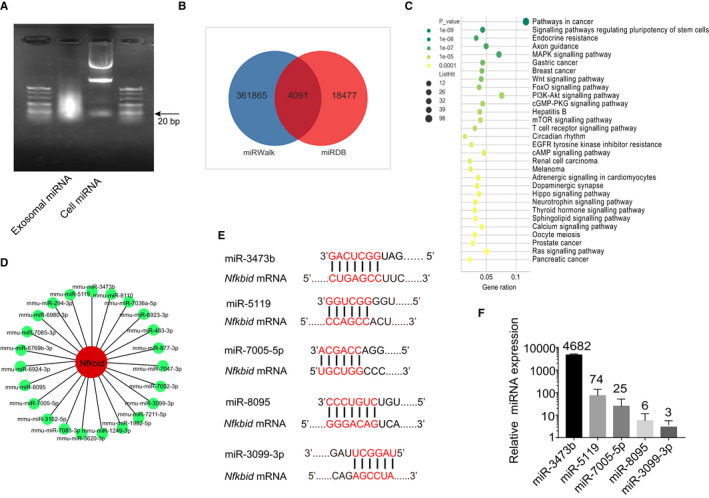
The identified miRNAs that could target the *Nfkbid*. A, Expression pattern of exosomal RNA and LLC‐derived RNA was shown by electropherogram. B, Microarray analysis identified 4091 miRNAs expression in LLC‐derived exosomes. C, Microarray analysis showed the signal pathways of these LLC‐derived exosomal miRNAs target genes. D, The interaction network of identified miRNAs that potentially targets *Nfkbid* gene. E, The binding sites between miRNAs and *Nfkbid* were shown. F, The expression of the five miRNAs with the highest relative expression was verified by qRT‐PCR. Each experiment was performed in triplicate

Next, we performed agarose nucleic acid electrophoresis of LLC‐derived exosomal RNA, and the exosomal RNA blow 30 bp are considered as miRNAs (Figure 3A). We further performed microarray analysis to generate miRNA profiles of LLC‐derived exosomes. Analysis of the two databases found that 4091 miRNAs were co‐expressed in LLC‐derived exosomes and predicted the signal pathways of these miRNAs target genes (Figure 3B,C). To investigate how LLC‐derived exosomes affect fibroblasts and promote lung metastasis, we performed RNA sequencing (RNA‐Seq) of LLC‐derived exosomes‐stimulated fibroblasts. Totally, 109 differentially expressed genes were identified (Figure S2A). KEGG pathway classification analysis found that the cluster of infectious diseases and immune system‐related genes were highly enriched (Figure S2B). LLC‐derived exosomes increased the expression of inflammatory response‐related genes, such as *Tl1*, and down‐regulated *Nfkbid* in fibroblasts, which were further confirmed by qRT‐PCR (Figure S2C). Interestingly, *Nfkbid* gene encodes NFKBID protein, which is a member of atypical inhibitor of NF‐κB by interacting with p50.^27^ Then, we screened twenty‐three miRNAs that could target *Nfkbid* (Figure 3D). Five miRNAs with the highest relative expression were selected, and the alignments between the sequences of those five miRNAs and the full length of the *Nfkbid* sequence were then determined, indicating that the *Nfkbid* coding sequences were their potential targets (Figure 3E). qRT‐PCR further verified the expression of those five miRNAs in LLC‐derived exosomes, among which miR‐3473b was the highest (Figure 3F).

### 3.4 LLC‐derived exosomal miR‐3473b induces the NF‐κB activation and inflammatory cytokines expression in fibroblasts

**Figure 4:**
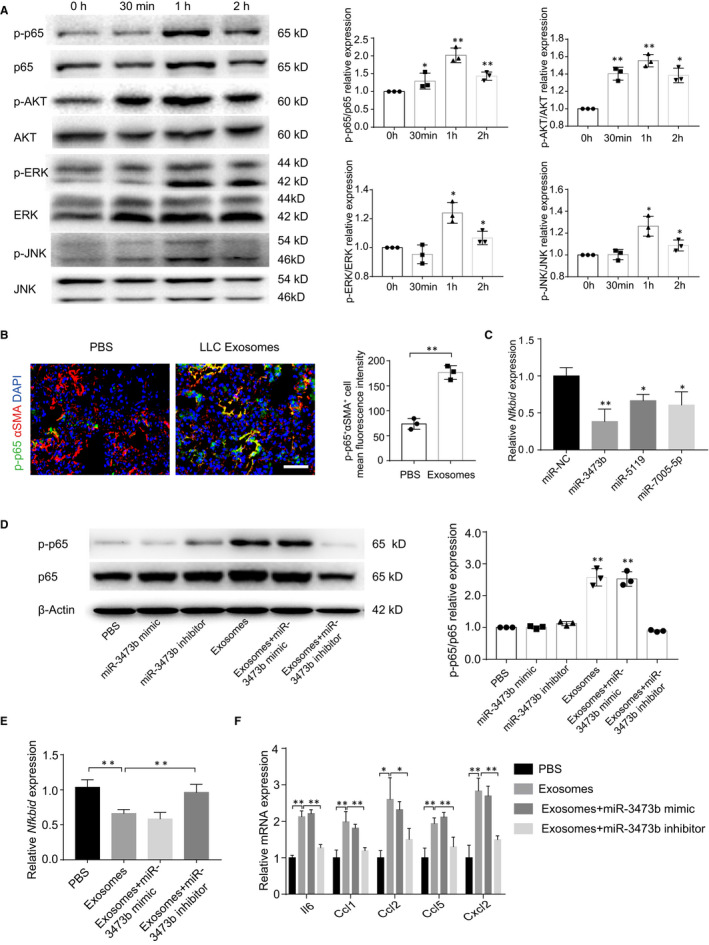
LLC‐derived exosomal miR‐3473b activated NF‐κB signalling pathway of fibroblasts. A, Western blot and densitometry analysis were used to determine and evaluated the expression of phosphorylated and total p65, AKT, ERK and JNK in fibroblasts, which stimulated with LLC‐derived exosomes in different time‐points. B, Immunofluorescence staining tested the expression of p‐p65 and αSMA in mouse lung tissues. p‐p65^+^αSMA^+^ cells were quantitatively analysed, and representative images were shown. Scale bar, 100 µm. Exposure times for all slides were optimized using the unfixed slide. C, The expression of *Nfkbid* in the miRNA mimic‐treated fibroblasts was detected by qRT‐PCR analysis. D, Western blot and densitometry analysis were used to verify and evaluated the expression of p‐p65 in the miR‐3473b mimic and inhibitor‐treated fibroblasts. E, The expression of *Nfkbid* in the miR‐3473b mimic and inhibitor‐treated fibroblasts was detected by qRT‐PCR analysis. F, The inflammatory genes in the miR‐3473b mimic and inhibitor‐treated fibroblasts were detected by qRT‐PCR analysis. Each experiment was performed in triplicate, and data are presented as mean ± SD Student's *t* test was used to analyse the data (*P < .05, **P < .01)

Next, we confirmed that LLC‐derived exosomes activated NF‐κB signalling pathway in fibroblasts, whereas AKT, ERK and JNK signalling pathways were slightly activated (Figure 4A). Other inflammatory genes such as *Il6, Ccl1, Ccl2, Ccl5* and *Cxcl2* were also increased (Figure S2C). Moreover, immunofluorescence results further confirmed that p‐p65 was highly expressed in αSMA + fibroblasts after 2 week's exosomes stimulation in vivo (Figure 4B). The above results show that LLC‐derived exosomes could activate the NF‐κB signalling pathway in fibroblasts by down‐regulating *Nfkbid*. To verify that LLC‐derived exosomal miRNAs could down‐regulate *Nfkbid*, we designed the mimics of miR‐3473b, miR‐5119 and miR‐7005‐5p. As expected, these miRNA mimics could reduce the expression of *Nfkbid* in fibroblasts, particularly miR‐3473b mimic (Figure 4C). Surprisingly, Exosomal miR‐3473b, not miR‐3473b mimic, could activate NF‐κB singling pathway, which suggests exosomal miR‐3473b activates the NF‐κB pathway is exosomes dependent. (Figure 4D). Further transfection experiments proved miR‐3473b inhibitor could abolish the exosomes induced NF‐κB activation in fibroblasts (Figure 4D, E). It has been demonstrated that NF‐κB is critical for tumour‐promoting inflammation which induced by tumour‐associated fibroblasts in many types of human cancer.^28^ Here, miR‐3473b inhibitor significantly reduced the exosome‐mediated inflammatory genes including *Il6, Ccl1, Ccl2, Ccl5* and *Cxcl2* expression in fibroblasts (Figure 4F), which suggest the potential role of exosomal miR‐3473b in establishing tumour‐promoting inflammation environment.

### 3.5 LLC‐derived exosomal miR‐3473b plays a major role in lung tumour cells colonization

**Figure 5:**
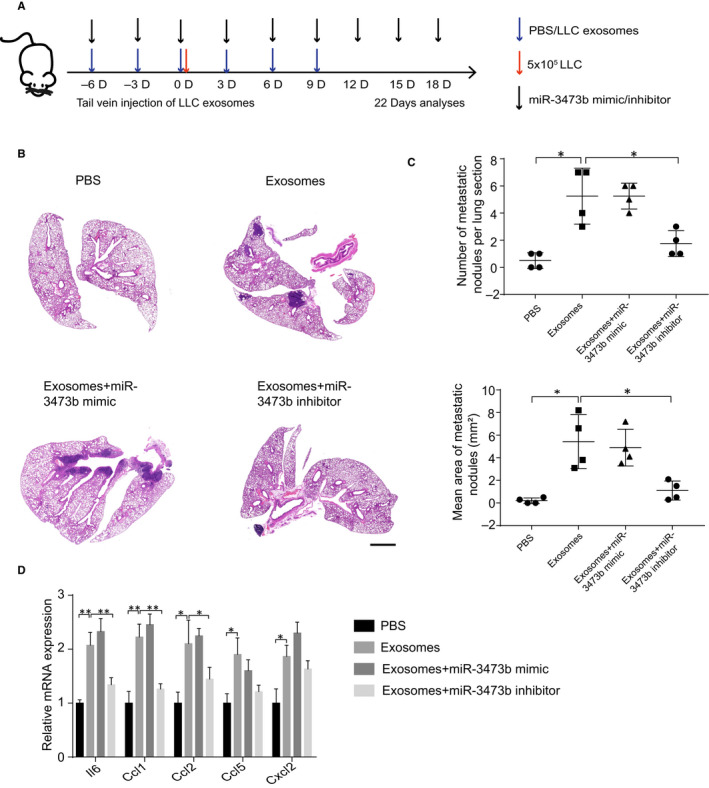
Exosomal miR‐3473b plays a major role in lung tumour cells colonization. A, Schematic diagram of exosomes or miR‐3473b inhibitor injection into the tail vein of mice (n = 4). B, Representative H&E images of experimental lung sections in three groups. Scale bar, 1000 µm. C, Summarized the numbers and areas of lung metastasis in three groups. D, qRT‐PCR was used to detect the inflammatory genes in the miR‐3473b mimic and inhibitor‐treated fibroblasts, which were sorted from the four treatment groups. Each experiment was performed in triplicate, and data are presented as mean ± SD Student's *t* test was used to analyse the data (*P < .05, **P < .01)

To detect the effect of miR‐3437b on lung tumour cells colonization, we injected LLC‐derived exosomes and exosomes mixed with miR‐3437b mimic or inhibitor into mice through the tail vein and, subsequently, injected LLC cells (Figure 5A). HE staining confirmed that the numbers and areas of lung metastasis in the miR‐3437b inhibitor‐treated mice were significantly reduced (Figure 5B,C). Moreover, miR‐3437b inhibitor treatment also reduced the B cells number in lungs (Figure S3A,B). To further investigate that the exosome induced LLC colonization was mediated by the increased inflammatory cytokines, we sorted lung fibroblasts from those four groups. RT‐PCR results showed *Il‐6* and *Ccl1* expression in miR‐3437b inhibitor‐treated group were clearly down‐regulated. Together, these data demonstrated that exosomal miR‐3473b could promote tumour cells colonization in the lung, which may relate to the inflammatory tumour‐promoting environment.

## 4 DISCUSSION

The intercellular communication in tumour microenvironment is responsible for tumour progression and metastasis. To clarify the mechanisms of exosome‐mediated crosstalk between tumour cells and stroma cells is very necessary. In this study, we firstly demonstrated that miR‐3473b, contained in the LLC‐derived exosomes, could activate the NF‐κB signalling pathway of lung fibroblasts and local inflammation, leading to enhanced intrapulmonary colonization of lung tumour cells. This provides a new mechanism of lung tumour‐derived exosomes induced tumour cells colonization by communicating with local fibroblasts.

As a new intracellular communication tool, exosomes play a vital role in tumour progression.^29^, ^30^ Studies indicate that primary tumour‐secreted exosomes could travel far from their original site and modify the local cells in the new metastatic site, and facilitate the primary tumour cells colonization in a second organ site.^31^, ^32^ For example, primary lung tumour‐derived exosomes could be absorbed by lung epithelial cells, leading to chemokine secretion and neutrophils recruitment, consequently resulting in lung PMN formation and lung metastasis.^33^ Our LLC‐derived exosomes tracking experiment suggested that more than 50% of pre‐injected exosomes were taken up by lung fibroblasts, whereas others were phagocytosed by immune cells. Although there have many different cell types in lung, the reason of fibroblasts and immune cells uptake majority of exosomes is not known yet.

It has been demonstrated that exosomes play key roles in fibroblasts activation, and the activated fibroblasts could promote tumour progression by producing inflammatory cytokines and growth factors. Goulet et al found that bladder tumour‐derived exosomes induce the bladder associated fibroblasts differentiate into pro‐tumour phenotype and secret high amount of pro‐inflammatory cytokine IL‐6.^34^ Gastric lung tumour‐derived exosomal miR‐27a converts fibroblasts to CAFs and promotes the proliferation, migration and metastasis of cancer cells both in vitro and in vivo.^35^ Here, our results suggested lung tumour‐derived exosomes promote the proliferation and inflammatory cytokines secretion in fibroblasts. Our RNA‐seq and exosomal microarray identified miR‐3473b was the most significant factor, which mediated the activation of NF‐κB in fibroblasts by targeting the *Nfkbid* gene. Of note, the miR‐3473b mimic cannot significantly up‐regulate the phosphorylation of p65, which suggests exosomal miR‐3473b activates the NF‐κB pathway is exosome‐dependent.

The function of miR‐3473b in tumour progression is not known yet. Wang et al reported that miR‐3473b was up‐regulated in mice brain following cerebral ischaemia and miR‐3473b antagomir could reduce the pro‐inflammatory gene expression in microglial,^19^ which indicates that miR‐3473b may have a role in the initiation of inflammation. Our miR‐3473b mimic and inhibitor‐based experiments revealed the function of miR‐3473b on NF‐κB activation and inflammatory cytokines expression in fibroblasts. Most importantly, inhibition of miR‐3473b could reverse lung tumour‐derived exosome‐mediated intrapulmonary colonization of lung tumour cells. Thus, our data indicate the pro‐inflammatory and tumour‐promoting function of miR‐3473b.

Increased chronic inflammation is a vital factor of tumour growth and metastasis.^36^ Lung pre‐metastatic niche‐derived pro‐inflammatory cytokines S100A8/9 could induce the secretion of serum amyloid A (SAA) 3, subsequently recruit Mac1^+^ macrophages and establish an inflammatory pre‐metastatic niche.^37^ Although we found miR‐3473b could induce the expression of pro‐inflammatory cytokines *Il6*, the mechanism of how these cytokines accelerate lung tumour cells colonization is still elusive. Interestingly, we found that B cell number in LLC‐derived exosomes injected mice lungs was significantly increased after lung tumour cells transplanted, and they were blocked by miR‐3473b inhibitor. The function of B cells remains controversial, particularly in tumour progression. Recent literatures illustrated the critical effects of B cells on immunotherapy responses, but the tumour‐promoting functions of B cells have been reported. For example, primary tumours induce B cell aggregation in draining lymph nodes, and these B cells selectively promote breast cancer lymph node metastasis through HSPA4‐targeting of IgG.^38^ In addition, B cell can also produce a variety of immunomodulatory cytokines that inhibit anti‐tumour immune responses by inhibiting effector cells such as CD8^+^ CTL and NK cells.^39^ Nevertheless, how LLC‐derived exosomes with or without LLC cells induced B cells accumulation and its role in lung tumour cell colonization remains to be further investigated.

Tumour seeding and colonization are the key paradigm for the initiation of metastasis. It has been documented that the systemic effects of tumour‐secreted factors and extracellular vesicles are the key elements for tumour cells colonization.^5^ Our results indicated that exosomes pre‐injection could active the local lung fibroblasts, induce its pro‐inflammatory cytokines secretion, and this might benefit for establishing an appropriate microenvironment for lung tumour cells seeding and outgrowth, the key step for tumour metastasis. Of note, to maintain the established tumour‐promoting environment, primary tumour cells continued to release exosome during the colonization of circulating tumour cells (CTCs).^40^ To mimic the existent of primary tumour cells, which could continuously release exosomes to blood circulation, we injected additional three times of exosomes to maintain the pro‐tumour seeding microenvironment after LLC exosomal pre‐injection and lung tumour cells transplantation. However, the disadvantage of the animal model is the numbers of exosome released from primary tumour cells are difficult to known and control, and more specific experiments need to be designed.

It is widely accepted that metastatic sites are not passive receivers of CTCs. The primary tumour prepared suitable environment for the seeding and outgrowth of CTCs by establishing PMN before metastatic spread.^41^ Studies are focused on understanding the pathological processes occurring before the development of PMN, wish to identify new targets to fight metastasis.^42^ Akoko et al found lung tumour cells could up‐regulate the expression of miR‐21 in lung fibroblasts, thus facilitate tumour progression by secreting calumenin.^43^ Myeloma cells reprogramme the bone marrow microenvironment by promoting the expression of miR‐27b‐3p and miR‐214‐3p in marrow fibroblasts.^44^ Those tumour‐derived miRNAs are potential targets for blocking the PMN and metastasis. Our results identified LLC‐secreted exosomal miR‐3473b could promote tumour cells colonization by modifying local fibroblasts’ behaviour, and targeting miR‐3473b could reduce the lung tumour cells seeding and outgrowth in lungs. However, the level of miR‐3473b in clinical lung tumour patients and its effect on lung fibroblasts still need to be further investigated.

In summary, this study indicates that the lung tumour‐derived exosomal miR‐3473b actives the NF‐kB pathway in local lung fibroblasts by targeting *Nfkbid* gene, leading to enhanced intrapulmonary colonization of lung tumour cells. Our study illustrates a new molecular mechanism of the communication between lung tumour cells and fibroblasts to facilitate lung tumour metastasis, providing a potential therapeutic target for lung cancer metastasis.

## CONFLICTS OF INTEREST

The authors declare no conflict of interest.

## AUTHOR CONTRIBUTIONS

**Cancan Du:** Data curation (lead); Formal analysis (lead); Investigation (lead); Software (lead); Visualization (lead); Writing‐original draft (lead). **Xixi Duan:** Methodology (equal). **Xiaohan Yao:** Methodology (equal). **Jiajia Wan:** Methodology (equal). **Yanru Cheng:** Methodology (supporting). **Yuan Wang:** Methodology (supporting). **Yan Yan:** Methodology (supporting). **LiJing Zhang:** Methodology (supporting). **Linyu Zhu:** Conceptualization (supporting). **Chen Ni:** Conceptualization (supporting). **Ming Wang:** Conceptualization (lead); Funding acquisition (equal); Investigation (equal); Project administration (lead); Supervision (equal); Validation (equal); Visualization (equal); Writing‐original draft (supporting); Writing‐review & editing (lead). **Zhihai Qin:** Conceptualization (equal); Funding acquisition (equal); Project administration (equal); Supervision (equal); Writing‐review & editing (supporting).

## Supporting information

Supinfo

## Data Availability

The microarray data were submitted to the Gene Expression Omnibus (GEO) database (accession number: GSE142584). The RNA sequencing data were uploaded in the Gene Expression Omnibus (GEO) database. The accession number is PRJNA597422. All other data supporting the findings of this study are available within the article and its Supporting Information files.
