# Supplementary material for: Tumour‐derived exosomal miR‐3473b promotes lung tumour cell intrapulmonary colonization by activating the nuclear factor‐κB of local fibroblasts
Source: J Cell Mol Med. 2020 May 25;24(14):7802–13. doi: 10.1111/jcmm.15411 (PMC7348150; doi:10.1111/jcmm.15411)
Supplement: Supplementary file 1 — Supinfo [file JCMM-24-7802-s001.docx]

**
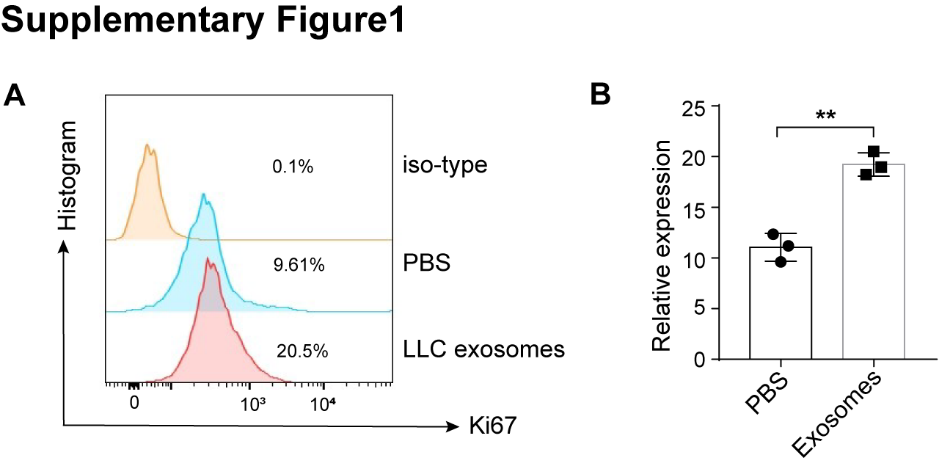
**

**Supplementary Figure.1.**

(A and B) Flow cytometry was used to detect the proliferation of LLC-derived exosomes-treated fibroblasts. The experiment was performed in triplicate, and data are presented as mean ± s.d. Student’s t-test was used to analyze the data (**P < 0.01).


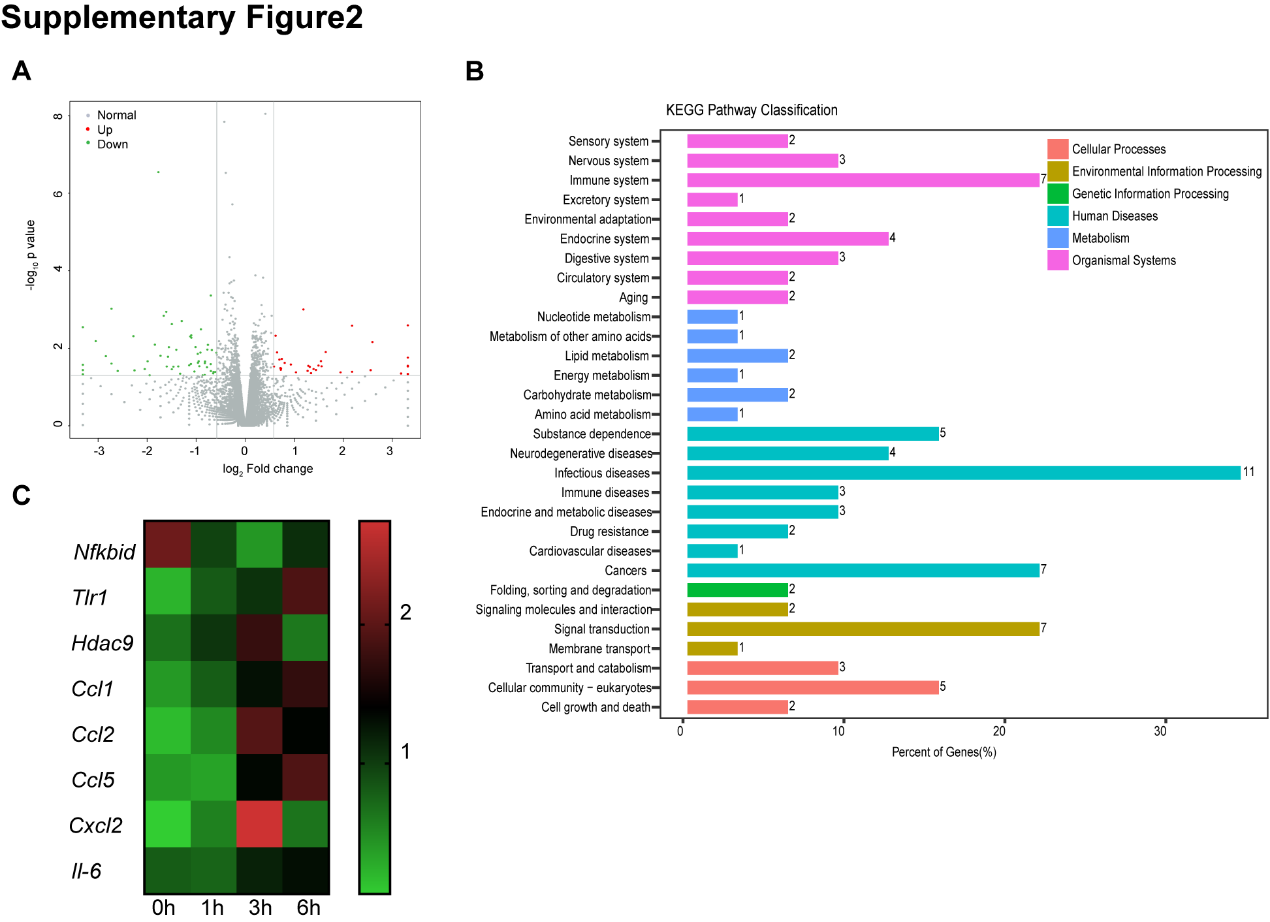


**Supplementary Figure.2.**

(A and B) RNA-Seq was performed to analyze genes expression in LLC-derived exosomes or PBS stimulated fibroblasts, the volcanic plot showing the differently expressed genes (A), the main KEGG pathways that are associated with the changed genes (B). (C) qRT-PCR verified inflammatory genes expression in fibroblasts which stimulated with LLC-derived exosomes in different time points.


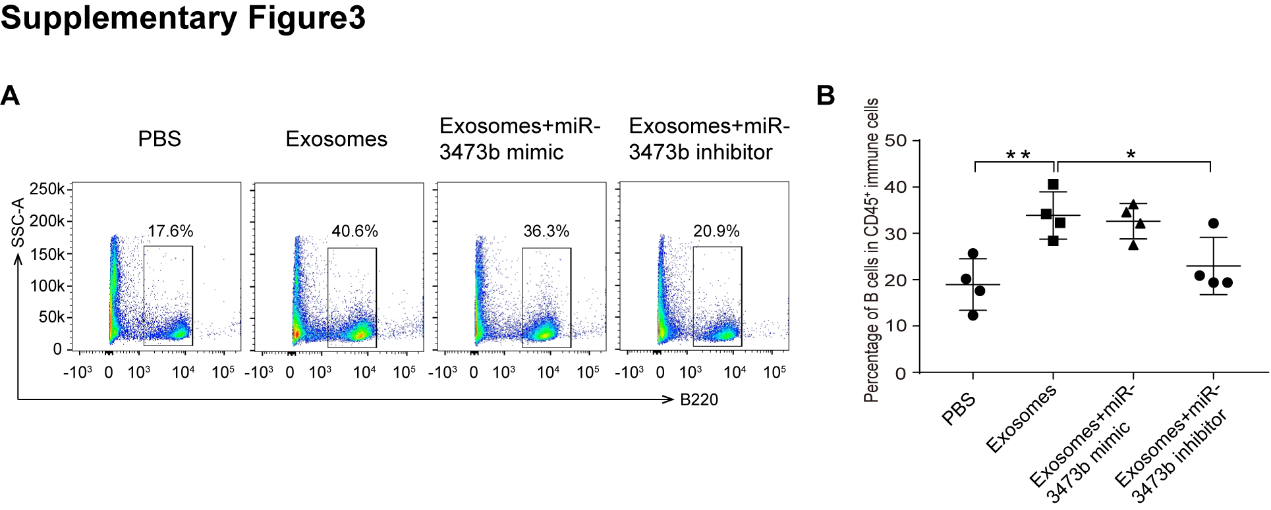


**Supplementary Figure.3.**

(A and B) The proportion of B cell in lung were detected by flow cytometry (n=4). Student’s t-test was used to analyze the data (*P < 0.05, **P < 0.01).

**Supplementary Table 1.** Details of antibodies.

| **Antibodies** | **Company** | **Lot No.** | [**concentration**](javascript:;) |
| --- | --- | --- | --- |
| CD45-Alexa Fluor700 | Biolegend | 103128 | 1:100 |
| FSP | Thermo Fisher | MA5-32347 | 1:100 |
| α-SMA | Abcam | ab21027 | 1:200 |
| CD63 | ABclonal | A5271 | 1:500 |
| Hsp90 | Cell Signaling Technology | 4877S | 1:1000 |
| Collagen type I | Proteintech | 14695-1-AP | 1:1000 |
| Fibronectin | Proteintech | 15613-1-AP | 1:1000 |
| p-p65 | Cell Signaling Technology | 3033 | 1:1000 |
| P65 | Cell Signaling Technology | 8042 | 1:1000 |
| p-AKT | Cell Signaling Technology | 4060 | 1:1000 |
| AKT | Cell Signaling Technology | 2920 | 1:1000 |
| p-ERK | Cell Signaling Technology | 4370S | 1:1000 |
| ERK | Cell Signaling Technology | 4695S | 1:1000 |
| p-JNK | Cell Signaling Technology | 4668S | 1:1000 |
| JNK | Cell Signaling Technology | 9258S | 1:1000 |
| B220-APC | Biosciences | 553092 | 1:100 |
| CD8-PerCP | Biolengend | 100732 | 1:100 |
| CD4-APC/Cy7 | Biolengend | 100413 | 1:100 |
| Ly-6G-APC | eBioscience | 17-5931-82 | 1:100 |
| CD11b-FITC | Biosciences | 553310 | 1:100 |
| Ki67 | Biolegend | 652401 | 1:100 |
| CD4 | Cell Signaling Technology | 25229 | 1:50 |
| CD8 | Cell Signaling Technology | 98941 | 1:200 |
| CD19 | Cell Signaling Technology | 90176 | 1:600 |
| Alexa Fluor 488 donkey anti-rabbit IgG | Thermo Fisher | A21206 | 1:400 |
| Alexa Fluor 488 donkey anti-goat IgG | Thermo Fisher | A11055 | 1:400 |

**Supplementary Table 2.**Sequences of primers.

| **Primer** | **Sequences** |
| --- | --- |
| *Nfkbid* | 5'-TCCAAGGATGCAGAATTCCC-3'  5'-GCTTGTATGACTGGCACCCAT-3' |
| *Tlr1* | 5'- AAGGTGATCTTGTGCCACCCAACAGTC -3'  5'- GATGGTGACAGTCAGCAGAACAGTATC-3' |
| *Hdac9* | 5'-CAGCAATAAGGAAAAGGCTGGG -3'  5'-GGTTGTTCACGTGGCAATGG -3' |
| *Ccl1* | 5'- CTCCACCAGACATTCGGCG -3'  5'- CATCCTGTATCCACACGGCA -3' |
| *Ccl2* | 5'- TTTTGTCACCAAGCTCAAGAGA -3'  5'- ATTAAGGCATCACAGTCCGAGT-3' |
| *Ccl5* | 5'- GCTGCTTTGCCTACCTCTCC-3'  5'- TCGAGTGACAAACACGACTGC -3' |
| *Cxcl2* | 5'- GCTTGAGTGTGACGCCCCCAGG -3'  5'- GTTAGCCTTGCCTTTGTTCAGTATC -3' |
| *Il6* | 5'-ACCCCAATTTCCAATGCTCTC -3'  5'-CATAACGCACTAGGTTTGCCG -3' |
| *Il10* | 5'-AAGGGTTACTTGGGTTGCCA -3'  5'-TTCAGCTTCTCACCCAGGGA -3' |
| *Fsp* | 5'- CACTTCCTCTCTCTTGGTCTGG-3'  5'- GTCACCCTCTTTGCCTGAGT -3' |
| *Acta2* | 5'- GTACCCAGGCATTGCTGACA -3'  5'- GCTGGAAGGTAGACAGCGAA -3' |
| *Fap* | 5'- GTGGAAGACAGACTTGCTTCTTT-3'  5'- TGTTCTGAAATCCAGTTGGGA -3' |
| *GAPDH* | 5'- AGGTCGGTGTGAACGGATTTG -3'  5'-TGTAGACCATGTAGTTGAGGTCA-3' |
| mmu-miR-3473b | 5'-GCGGGCTGGAGAGATG -3'  5'-GTGCAGGGTCCGAGGT -3' |
| mmu-miR-5119 | 5'-GCGCCATCTCATCCTGG -3'  5'-GTGCAGGGTCCGAGGT -3' |
| mmu-miR-8095 | 5'-GCGCAAAGGATTCTGCTGTC -3'  5'-GTGCAGGGTCCGAGGT -3' |
| mmu-miR-3099-3p | 5'-GCGCTAGGCTAGAGAGAGGT -3'  5'-GTGCAGGGTCCGAGGT -3' |
| mmu-miR-7005-5p | 5'-CCTGGGGATGGGAGGA -3'  5'-GTGCAGGGTCCGAGGT -3' |
| U6 | 5'-CTCGCTTCGGCAGCACA -3'  5'-AACGCTTCACGAATTTGCGT -3' |

**Supplementary Table 3.**Sequences of miRNA mimics.

| **Primer** | **Sequences** |
| --- | --- |
| mmu-miR-3473b | 5'-GGGCUGGAGAGAUGGCUCAG -3'  5'-CUGAGCCAUCUCUCCAGCCC -3' |
| mmu-miR-5119 | 5'-CAUCUCAUCCUGGGGCUGG-3'  5'-CCAGCCCCAGGAUGAGAUG-3' |
| mmu-miR-7005-5p | 5'-CCUGGGGAUGGGAGGACCAGCA-3'  5'-UGCUGGUCCUCCCAUCCCCAGG-3' |
| mimics-nc | 5'-UUCUCCGAACGUGUCACGUTT -3'  5'-ACGUGACACGUUCGGAGAATT-3' |

**Supplementary Table 4.**Sequences of miRNA inhibitor.

| **Primer** | **Sequences** |
| --- | --- |
| mmu-miR-3473b | 5'-UGCUGGUCCUCCCAUCCCCAGG-3' |
| inhibitor-nc | 5'-CAGUACUUUUGUGUAGUACAA-3' |

**Abbreviations:** NFKBID, NFKB inhibitor delta; MPLC, multiple primary lung cancer; MIF, migration inhibitory factor; SOCS3, suppressor of cytokine signaling 3; MCAO, middle cerebral artery occlusion; CAF, cancer-associated fibroblast; HCC, Hepatocellular carcinoma; NF-κB, nuclear factor-κB; LLC, Lewis lung carcinoma; FSP, fibroblast specific protein; α-SMA, α-smooth muscle actin;
